# Importance of Coverage and Endemicity on the Return of Infectious Trachoma after a Single Mass Antibiotic Distribution

**DOI:** 10.1371/journal.pntd.0000507

**Published:** 2009-08-25

**Authors:** Takele Lakew, Wondu Alemayehu, Muluken Melese, Elizabeth Yi, Jenafir I. House, Kevin C. Hong, Zhaoxia Zhou, Kathryn J. Ray, Travis C. Porco, Bruce D. Gaynor, Thomas M. Lietman, Jeremy D. Keenan

**Affiliations:** 1 Orbis International, Addis Ababa, Ethiopia; 2 F. I. Proctor Foundation, University of California, San Francisco, California, United States of America; 3 Department of Ophthalmology, University of California, San Francisco, California, United States of America; 4 Department of Epidemiology & Biostatistics, University of California, San Francisco, California, United States of America; 5 Institute for Global Health, University of California, San Francisco, California, United States of America; Weill Medical College of Cornell University, United States of America

## Abstract

**Background:**

As part of the SAFE strategy, mass antibiotic treatments are useful in controlling the ocular strains of chlamydia that cause trachoma. The World Health Organization recommends treating at least 80% of individuals per community. However, the role of antibiotic coverage for trachoma control has been poorly characterized.

**Methodology/Principal Findings:**

In a collection of cluster-randomized clinical trials, mass oral azithromycin was administered to 40 villages in Ethiopia. The village prevalence of ocular chlamydia was determined before treatment, and at two and six months post-treatment. The mean prevalence of ocular chlamydia was 48.9% (95% CI 42.8 to 55.0%) before mass treatments, decreased to 5.4% (95% CI 3.9 to 7.0%) at two months after treatments (*p*<0.0001), and returned to 7.9% (95% CI 5.4 to 10.4%) by six months after treatment (*p* = 0.03). Antibiotic coverage ranged from 73.9% to 100%, with a mean of 90.6%. In multivariate regression models, chlamydial prevalence two months after treatment was associated with baseline infection (*p*<0.0001) and antibiotic coverage (*p* = 0.007). However, by six months after treatment, chlamydial prevalence was associated only with baseline infection (*p*<0.0001), but not coverage (*p* = 0.31).

**Conclusions/Significance:**

In post-hoc analyses of a large clinical trial, the amount of endemic chlamydial infection was a strong predictor of chlamydial infection after mass antibiotic treatments. Antibiotic coverage was an important short-term predictor of chlamydial infection, but no longer predicted infection by six months after mass antibiotic treatments. A wider range of antibiotic coverage than found in this study might allow an assessment of a more subtle association.

## Author Summary

Trachoma, caused by ocular chlamydia infection, is the most common infectious cause of blindness in the world. The World Health Organization (WHO) recommends the SAFE strategy (eyelid surgery, antibiotics, facial hygiene, environmental improvements) for trachoma control. Oral antibiotics reduce the transmission of ocular chlamydia, but re-infection of treated individuals is common. Therefore, the WHO recommends annual mass antibiotic treatments to the entire village. The success of treatment is likely based on many factors, including the antibiotic coverage, or percentage of villagers who receive antibiotics. However, no studies have analyzed the importance of antibiotic coverage for the reduction of ocular chlamydia. Here, we performed multivariate regression analyses on data from a clinical trial of mass oral antibiotics for trachoma in a severely affected area of Ethiopia. At the relatively high levels of antibiotic coverage in our study, coverage was associated with post-treatment infection at two months, but not at six months. The amount of infection at baseline was strongly correlated with post-treatment infection at both two and six months. These results suggest that in areas with severe trachoma treated with relatively high antibiotic coverage, increasing coverage even further may have only a short-term benefit.

## Introduction

The World Health Organization (WHO) recommends the SAFE strategy (eyelid surgery, mass antibiotics, facial hygiene promotion, and environmental improvement) for the control of trachoma, the world's leading infectious cause of blindness[1]. Mass antibiotic treatments target the ocular strains of chlamydia that cause trachoma, and are a crucial component of the SAFE strategy. A single dose of oral azithromycin is clearly effective in eliminating infection from individual cases[2],[3]. A mass distribution of azithromycin to an entire community has been shown to dramatically reduce the prevalence of infection. Unfortunately, infection returns in areas with hyper-endemic trachoma[4]–[6]. Theoretically, repeated treatments can progressively reduce the prevalence of, and even eliminate, infection[7]. However, models suggest that in severely affected areas, treatment would have to be given frequently and to a large portion of the population[8],[9].

There have been few studies examining the role of antibiotic coverage for trachoma control, aside from the observation that mass antibiotic treatments with high coverage have resulted in a considerable reduction in ocular chlamydia prevalence, and even elimination of infection[10],[11]. Many think that low antibiotic coverage may play a crucial role in persistent ocular chlamydial infection[12]. Currently, the WHO recommends a goal of 80% antibiotic coverage for trachoma programs[1]. However, the relationship between antibiotic coverage and treatment efficacy at the community level has not been well characterized. Mathematical models have suggested that at higher antibiotic coverages, less frequent mass treatments will be required, and elimination will occur in a shorter period of time[7],[9]. It is not clear what level of coverage will be necessary, or whether different guidelines will be necessary for more severely affected areas[5],[8]. Here we assess how the prevalence of infection two and six months post-treatment is dependant on the antibiotic coverage and the amount of endemic infection at baseline.

## Methods

The Committee on Human Research at the University of California, San Francisco approved this post-hoc analysis of existing data, and approved the use of verbal informed consent, which was obtained by local Amharic-speaking health workers from all study participants at the time of each procedure. Verbal consent was performed due to the high amount of illiteracy in the region.

As part of a larger, multiple arm, group-randomized trial, 40 villages were enrolled in the Gurage Zone of southern Ethiopia[6],[7],[13],[14]. Although the 40 villages were distributed between five study arms, they received identical treatment and monitoring for the initial six months, the results of which are reported here. An initial census of the study area was performed by trained local health workers; the names of all permanent residents in each village were recorded. The population of villages ranged from 122 to 976, with a median of 368 persons (interquartile range 243 to 502). Those aged 1 year and older were offered a single dose of directly observed, oral azithromycin (1g in adults or 20 mg/kg in children). Pregnant women and those allergic to macrolides were offered a six-week course of topical 1% tetracycline ointment (applied twice daily to both eyes and not directly observed). Antibiotic coverage was defined as the proportion of permanent residents eligible for treatment in the village (i.e., those ≥1 year of age) who accepted directly observed treatment with oral azithromycin or not-directly observed topical tetracycline, as determined from the baseline census by the health workers who distributed the antibiotics. Individuals known to have either moved permanently or died between the census and the scheduled treatment were not included in the denominator. Children aged 1–5 years were monitored at baseline, two months, and six months post-treatment, as described below. After the six month monitoring, some communities received biannual treatment, some annual treatment, and some no further treatment unless infection surpassed a pre-assigned level. The results of these trials are published elsewhere[6],[7],[13],[14].

All children aged 1–5 years in treated villages were assessed for the presence of ocular chlamydia infection at baseline (pre-treatment), two months post-treatment, and six months post-treatment. A dacron swab was passed firmly across the right upper tarsal conjunctiva three times, rotating between each pass. Examiners wore new gloves for each study subject. All samples were kept at 4°C in the field and frozen at −20°C within six hours. The swabs were shipped at 4°C to San Francisco where they were stored at −70°C until processed. The AMPLICOR PCR test (Roche Diagnostics, Branchburg, NJ) was used to detect chlamydial DNA. Pre-treatment samples were tested individually, and post-treatment samples were analyzed as pooled samples. PCR pooling is a well established, cost-effective technique for diagnosis of genital and ocular chlamydia[15],[16]. Post-treatment samples from the same village were randomized and pooled into groups of 5, with a possible remainder pool of 1–4 samples. Each pool was then tested according to the AMPLICOR protocol. If two-thirds or more of the pools were positive, the individual samples were re-pooled randomly into groups of two and re-processed to allow more accurate estimation[16]. If PCR of any pool was equivocal, then all samples from the pool were individually re-tested. As per the AMPLICOR protocol, an internal control was performed for each pool to rule out the presence of PCR inhibitors. Any inhibitory pools were re-tested, and if still inhibitory, the samples were tested individually. While samples necessarily were diluted in the pooling process, this is not thought to significantly impact the sensitivity of the test[17]. The prevalence of ocular chlamydia infection in each village was obtained by maximum likelihood estimation[7]. The number of positive individual samples most likely to have resulted in the observed pooled PCR results was chosen as the estimate for that village (Mathematica 5.0, Wolfram Research Inc., Champaign, IL).

All statistical analyses were conducted at the village level using village prevalence; no individual-level data was used in this study. This is appropriate, since trachoma interventions occur at the village level, and treatment success is measured at the village level, not at the individual level. The distribution of the prevalence of antibiotic coverage was depicted with a kernel density plot, using the Epanechnikov kernel function, the Sheather-Jones plug-in bandwidth estimate, and upper boundary correction using the renormalization method. The Wilcoxon signed rank test was used to compare the prevalence of infection at baseline with two months, and at two months with six months. The Spearman rank order correlation coefficient and 95% confidence interval was calculated for pairwise combinations of baseline infection, antibiotic coverage, infection at two months after treatment, and infection at six months after treatment. Multivariate regression was performed to assess the relationship between the prevalence of infection post-treatment with antibiotic coverage and endemic (baseline) infection. Linear regression models were constructed using the prevalence of chlamydial infection at either two or six months as the response variable, and baseline prevalence of infection and antibiotic coverage as explanatory variables, using the robust variance calculation based on the HC3 heteroskedasticity consistent covariance matrix estimator, due to concerns about heteroskedastic residuals[18]. Infection prevalence at all time points was square-root transformed to minimize heteroskedasticity and maximize normality of the residuals from the linear regression analysis (analyzed by plotting the residuals vs. the fitted values and residuals vs. the predictors; in addition, no heteroskedasticity was demonstrated with the Cook-Weisberg test, and no departure from normality was observed with the Shapiro-Francia test, using a significance level of 0.05). Linearity of the predictors in the model was adequate, as assessed with component-plus-residual plots comparing the linear fit of the predictor to the LOWESS curve. Multivariate regression models were constructed including the multiplicative interaction term for antibiotic coverage and baseline infection, but interaction terms were not significant, and therefore not included in the final model. All statistical analyses were performed with STATA 10.0 (Statacorp, College Station, TX).

## Results

**Figure 1:**
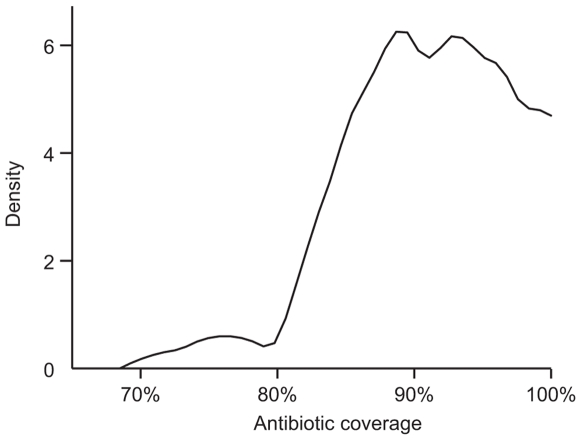
Kernel density estimate showing the distribution of antibiotic coverage. Antibiotic coverage data was available for 38 of 40 villages. The density plot was computed using the Epanechnikov kernel function, Sheather-Jones plug-in bandwidth estimate, and upper boundary correction using the renormalization method.

The mean number of children ages 1–5 examined in each village at baseline was 54.2 (95% CI 45.7 to 62.8). No villages were lost to follow up. The mean pre-treatment prevalence of infection in 1–5 year old children among the 40 study villages was 48.9% (95% CI 42.8 to 55.0%). Antibiotic coverage data was present for 38 of the study villages, and ranged from 73.9% to 100%, with a mean of 90.6% (95% CI 88.7 to 92.4%). As is evident in a density plot, the majority of villages had an antibiotic coverage between 80–100% (Figure 1). Two months after treatment, infection decreased significantly from baseline, to a mean of 5.4% (95% CI 3.9 to 7.0%), *p*<0.0001. Between two and six months after treatment, the village prevalence of infection increased, to a mean of 7.9% (95% CI 5.4 to 10.4%), *p* = 0.03, compared to two months).

Using Spearman's test of correlation, prevalence of infection in 1–5 year old children at two months was strongly correlated with baseline infection (*r_s_* = 0.62, 95% CI 0.39 to 0.78), and moderately correlated with antibiotic coverage (*r_s_* = −0.31, 95% CI −0.58 to 0.01). The prevalence of infection in 1–5 year old children at six months was strongly correlated with infection at baseline (*r_s_* = 0.55, 95%CI 0.28 to 0.73) and at two months (*r_s_* = 0.73, 95% CI 0.54 to 0.85), but only weakly correlated with antibiotic coverage (*r_s_* = −0.16, 95% CI −0.46 to 0.17).

**Figure 2:**
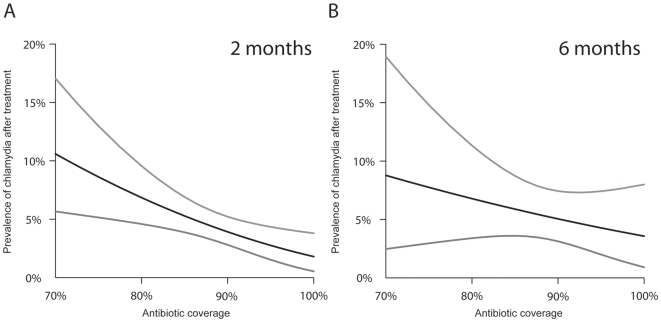
Predicted chlamydial infection after a single mass azithromycin treatment, with varying levels of antibiotic coverage. Post-treatment chlamydial prevalence in 1–5 year old children was calculated for a hypothetical community treated with a single mass azithromycin treatment, in which 48.9% of 1–5 year old children were infected at baseline. Antibiotic coverage was significantly associated with post-treatment infection at two months (2A; *R* ^2^ = 0.53, *p* = 0.007), but not at six months (2B; *R* ^2^ = 0.35, *p* = 0.31). The upper and lower curves are the boundaries of the 95% confidence interval for the predicted mean.

Multivariate regression models demonstrated that at two months after treatment, chlamydial infection in 1–5 year old children was predicted by both baseline chlamydial infection and antibiotic coverage (*R* ^2^ = 0.53, Table 1). By six months after treatment, baseline chlamydial infection remained a significant predictor of chlamydial infection, but antibiotic coverage did not (*R^2^* = 0.35, Table 1). Because the square root transformation of the response variable made the regression coefficients difficult to interpret, we used the models to calculate the role of antibiotic coverage in predicting post-treatment chlamydia in a hypothetical community, holding the baseline prevalence of infection constant at 48.9% (the mean baseline infection in this study). As depicted in Figure 2, antibiotic coverage had a greater effect in predicting chlamydial prevalence at two months compared to six months, evident from the steeper curve and narrower 95% confidence intervals.

**Table 1:** Multivariate regression models analyzing the prevalence of baseline infection and antibiotic coverage as predictors for the prevalence of chlamydial infection at two months and 6 months after treatment.

Response variable	Explanatory variable	*β*(SE)	*p*-value	*R* ^2^
Infection at two months	Baseline infection	0.59 (0.10)	<0.0001	0.53
	Antibiotic coverage	−0.64 (0.22)	0.007	
Infection at six months	Baseline infection	0.70 (0.13)	<0.0001	0.35
	Antibiotic coverage	−0.36 (0.35)	0.31	

Regression analyses used HC3 robust variance calculation, with the square root transformation of chlamydial infection at baseline, two months post-treatment, and six months post-treatment.

## Discussion

Mathematical models and clinical trials have demonstrated the importance of vaccine coverage for conveying immunity on a population[19],[20]. Analogously, antibiotic coverage has been touted as an important determinant in the long-term success of the WHO's mass antibiotic treatments for trachoma[5],[7],[9],[21],[22]. Some have suggested that a single mass antibiotic treatment may prevent infection from returning, if given to a sufficiently large proportion of the community[23],[24]. In this study, coverage was important at two months after treatment, but we were unable to demonstrate its importance at six months.

In our study, baseline infection was a significant predictor of chlamydial infection at both two months and six months, whereas antibiotic coverage predicted chlamydial infection only at two months. Thus, in this severely affected area, the amount of endemic infection appears to be a stronger determinant of chlamydial infection than antibiotic coverage. This may be the case for at least three reasons. First, communities with more initial infection will tend to have more residual infection after an incomplete mass treatment, as demonstrated by mathematical models[8]. Secondly, re-infection after mass treatments likely occurs more rapidly in areas with severe trachoma, due to underlying transmission characteristics in these areas, such as poor hygiene and sanitation, travel to untreated communities, genetic variation among chlamydial strains and a myriad of other risk factors[25]–[27]. This suggests that in the long run, the forces that result in the return of ocular chlamydia into severely affected communities may overwhelm any temporary advantage conferred by high antibiotic coverage[26],[28],[29]. Third, children under 1 year of age were not treated with oral antibiotics in this study, and may therefore have served as a reservoir for infection. It is possible that the level of endemic infection is an indicator of infection in these untreated children. If so, then our finding of a strong relationship between endemic infection and post-treatment infection may indicate that chlamydial transmission from this young age group was more important than antibiotic coverage in predicting the prevalence of chlamydial infection after treatment.

If chlamydial infection does depend more on endemic infection than antibiotic coverage, this would not support devoting more resources to increasing the target antibiotic coverage from WHO guidelines, which currently recommend 80% antibiotic coverage. It is important to note that this regression model should not be extrapolated outside the range of our data; therefore, this conclusion may be generalizable only to severely affected areas with relatively high coverage. It is possible that antibiotic coverage may carry more importance in areas with milder trachoma and slower return of chlamydia after mass treatments.

This analysis supports the theory that treatment frequency and duration could be tailored to areas based on pre-treatment prevalence. Currently, the WHO recommends three annual mass treatments to trachoma-endemic areas, with re-evaluation after the third treatment[1]. This strategy has proven very successful in nearly eliminating infection in an area with a modest-moderate amount of trachoma[30]. However, fewer treatments may be sufficient in communities with hypoendemic trachoma and low pre-treatment chlamydial prevalence[24]. In contrast, communities with hyperendemic trachoma and high pre-treatment chlamydial prevalence may require a greater number of treatments, or more frequent treatments, as suggested by mathematical models[7]–[9],[13] and clinical trials[13],[14],[31]. In this study, chlamydial prevalence after mass antibiotic treatments was strongly predicted by the pre-treatment prevalence of infection, which supports the idea that villages could be stratified by pre-treatment chlamydial prevalence and offered a tailored mass antibiotic treatment regimen. Further research is needed to determine whether this could be a feasible strategy.

Few previous studies have addressed the question of antibiotic coverage for ocular chlamydia, aside from noting the success of mass antibiotic efforts with high coverage[10],[11],[32]. However, our findings are consistent with a study of mass azithromycin in multiple villages in the Gambia[29]. In the Gambian study, re-emergence of chlamydial infection in a subset of villages could not be explained by antibiotic coverage, which averaged 83% among the villages. These findings are consistent with the possibility that at the 80% antibiotic coverage target currently recommended by the WHO, other predictors become more important than differences in coverage.

There are several limitations of this study. We focused on one component of the SAFE strategy: mass antibiotic distributions, and are unable to comment on the role of antibiotic coverage in the setting of other trachoma interventions. We analyzed predictors of chlamydial infection at the community level, and therefore cannot make any conclusions regarding individuals in the community. However, individual-level data are not particularly helpful for trachoma programs, which make treatment decisions based on community indicators of trachoma. There was a relatively short surveillance time of six months, at which point some of the communities had scheduled re-treatments. Migration in the study area was not studied. The range of antibiotic coverage was relatively narrow, which may have decreased the likelihood of finding a significant effect of antibiotic coverage in the regression models, and may decrease the generalizability of the study. Finally, since communities in this study were not randomized to a pre-specified antibiotic coverage, unmeasured confounders may have affected the regression analyses. We would expect, however, that these confounders would have biased toward an association between antibiotic coverage and infection, since those communities where it is difficult to attain high antibiotic coverage may also have conditions that enhance the transmission of ocular chlamydia.

In conclusion, in post-hoc analyses of a large clinical trial of mass azithromycin for trachoma, we found that antibiotic coverage predicted chlamydial infection at two months after treatment, but not at six months after treatment. Far more important was baseline chlamydial infection, which was a strong predictor of infection at both two months and six months. This suggests that the WHO's recommendation of 80% antibiotic coverage is reasonable, and that trying to further increase antibiotic coverage may be less successful than targeting more intensive treatments to highly prevalent communities. Clinical trials in which communities are randomized to different antibiotic coverage levels will be important to more fully characterize the relationship between antibiotic coverage and chlamydial infection after mass antibiotic treatments.
